# The research interest, capacity and culture of NHS staff in South East Scotland and changes in attitude to research following the pandemic: a cross-sectional survey

**DOI:** 10.1186/s12913-023-09196-y

**Published:** 2023-03-07

**Authors:** David J Chinn, Magdalena Pribanova, Frances Quirk

**Affiliations:** 1 Research, Innovation and Knowledge Department, NHS Fife, Queen Margaret Hospital, Whitefield Road, Dunfermline, Fife, KY12 0SU Scotland; 2 Fife Psychology Service, Psychology Department, NHS Fife, Lynebank Hospital, Halbeath Road, Dunfermline, Fife, KY11 4UW Scotland

**Keywords:** Research Capacity, Health Research, Staff Development, Research Capacity Development, Innovation, Research Activity, Barriers, Motivators, Pandemic

## Abstract

**Background:**

The UK National Health Service (NHS) is ideally placed to undertake research. The UK Government recently launched its vision of research within the NHS to improve research culture and activity amongst its staff. Currently, little is known about the research interest, capacity and culture of staff in one Health Board in South East Scotland and how their attitudes to research may have changed as a result of the SARS-CoV-2 pandemic.

**Methods:**

We used the validated Research Capacity and Culture tool in an online survey of staff working in one Health Board in South East Scotland to explore attitudes to research at the organisation, team and individual level together with involvement in, barriers to and motivators to engage in research. Questions included changes in attitude to research as a result of the pandemic. Staff were identified by professional group: nurses/midwives, medical/dental, allied health professionals (AHP), other therapeutic and administrative roles. Median scores and interquartile ranges were reported and differences between groups assessed using the Chi-square and Kruskal-Wallis tests with P < 0.05 accepted as statistical significance. Free-text entries were analysed using content analysis.

**Results:**

Replies were received from 503/9145 potential respondents (5.5% response), of these 278 (3.0% response) completed all sections of the questionnaire. Differences between groups were noted in the proportions of those with research as part of their role (P = 0.012) and in being research-active (P < 0.001). Respondents reported high scores for promoting evidence-based practice and for finding and critically reviewing literature. Low scores were returned for preparing reports and securing grants. Overall, medical and other therapeutic staff reported higher levels of practical skills compared with other groups. Principal barriers to research were pressure of clinical work and lack of time, backfill and funds. 171/503 (34%) had changed their attitude to research as a result of the pandemic with 92% of 205 respondents more likely to volunteer for a study themselves.

**Conclusion:**

We found a positive change in attitude to research arising from the SARS-CoV-2 pandemic. Research engagement may increase after addressing the barriers cited. The present results provide a baseline against which future initiatives introduced to increase research capability and capacity may be assessed.

**Supplementary Information:**

The online version contains supplementary material available at 10.1186/s12913-023-09196-y.

## Background

The UK National Health Service (NHS) is well placed to engage in clinical research and makes a significant contribution to the international evidence base improving treatments and outcomes for patients. In 2021 the UK Government published its ‘Vision for Research’ [1] and implementation plan 2022–2025 [2] to ensure that clinical research is firmly embedded in the NHS by increasing the research culture amongst all its health care staff. This vision has been influenced by the lessons learned from the SARS-CoV-2 pandemic in which the NHS was instrumental in delivering the research needed to identify effective treatments, for example, through the RECOVERY trial [3], and in contributing to the global effort to develop suitable vaccines.

One approach in the Government’s vision is to empower all clinical staff to consider the importance of research in improving patient pathways and to encourage them to view research as part of their role. An initial approach is to assess staff attitudes to research, the culture within the service and the motivation and capacity to actively engage in research. Previous studies have revealed substantial differences in attitudes and research capacity between diverse professional groups [4, 5]. For example, nurses and allied health professionals (AHPs) appear less confident and cite fewer skills than medical practitioners in engaging with research. Strategies proposed for building research capacity in health care settings have been directed at overcoming barriers to engagement in research including, for example, the provision of research training [6], short-term funding to provide full-time back fill for clinicians [7], protected time [4, 8] and mentors to support novice researchers in their early careers [9]. Other approaches have included establishing multi-centred research networks [10] and engagement of dedicated research facilitators within the organisation [11–13].

The NHS in Scotland comprises 14 separate Health Boards of varying size and engagement in research activities aligned to the Scottish Government’s Research Strategy [14]. NHS Fife serves a population of about 350,000 and has a small, active pool of researchers supported by a Research, Innovation and Knowledge department comprising three teams: Research and Development, Innovation and Library and Knowledge services. Staff include clinical research nurses, librarians, research advisers and administrative staff dealing with, for example, research governance and finance. However, knowledge of the attitudes to, and interest in research is scarce amongst the main workforce. We have surveyed the staff to assess the current research culture and elicit their interest in and attitudes to research with the intention of developing initiatives to increase overall research capacity in line with the UK and Scottish Government’s visions [1, 14]. The results provide a baseline against which change in activity will be measured to evaluate the effectiveness of the strategies introduced. The aims of the study were to explore the research interest, capacity and culture among diverse staff groups, to identify personal and organisational barriers and motivators in engaging in research and to determine the impact of the pandemic on staff attitudes to research in the NHS.

## Methods

We used a mixed methods approach with an anonymous, online survey open to all staff working for NHS Fife and the Health and Social Care Partnership, together with semi-structured interviews with volunteer staff to obtain more in-depth information on barriers, motivators, attitudes and opportunities to engage in research. A validated questionnaire, the Research Capacity and Culture tool (RCCT) was used in the online survey supplemented with additional questions on staff attitudes to research and how these may have changed as a result of the pandemic. The RCCT of Holden et al. [15] includes questions on the respondent’s demographics, current and previous engagement in research, and aspects of research at three levels: organisation, team and individual, with these aspects scored on a Likert scale of 1–10, with 1 indicating the lowest level of skill / success and 10 indicating the highest level of skill / success. An additional option of ‘Unsure’ was included. Additional questions covered personal barriers and motivators to engaging in research with an option for free-text entries. The RCCT uses the responses to this set of questions to describe research culture, reflecting the environment and support available to researchers, and research capacity, reflecting the skills and abilities of individuals within the organisation to conduct meaningful, quality assured research. The survey was advertised widely on staff computer desktop screens and via the NHS Fife Communication Department’s weekly updates, which are sent to all those with an NHS Fife email address, and delivered using Smart Survey®. Managers were asked to encourage their staff to participate and promote the survey within team meetings. Respondents were offered the chance to be included in a prize draw to win a £100 gift voucher. Personal contact details were collected in a separate data collection scheme for those wishing to be included in the prize draw and for those volunteering for the semi-structured interviews; email addresses were not linked to their survey responses to maintain anonymity. The survey was undertaken between November 2021 and January 2022 with regular reminders issued via staff notices. A realistic target sample size was set as 5% of the current workforce of 9,145. Respondents to the survey could also indicate their willingness to participate in a semi-structured interview, the results of which will be reported in a separate communication.

NHS management approval was obtained but a formal review by an NHS ethics committee was not required as the research participants were NHS staff only. Completion of the survey was taken as implied consent.

Data were analysed using SPSS (version 26). Simple descriptive summary measures of the responses of staff were split according to their professional grouping (Medical and Dental, Nursing and Midwifery, Allied Health Professionals (AHPs), Other therapeutic staff and Admin/support staff). Medians and inter-quartile ranges were reported as the data were non-parametric in distribution. Further analyses investigated the magnitude and statistical significance of differences in summary scores between different professional groups using the Chi-square test and Kruskal-Wallis test. A significance level of P < 0.05 (two-tailed) was adopted to indicate statistical significance. The Bonferroni correction was applied to reduce spurious findings arising from multiple comparisons. Free-text entries were analysed using content analysis by all the authors.

## Results

Overall, replies were obtained from 503 members of staff (response rate 5.5%), with 278 (3.0% response) completing all sections of the questionnaire, including the demographic section. Of the latter, 214 (78%) were females, 50 (18%) were males and 10 (4%) preferred not to answer the question. Of those who provided their age 37% were aged 50 years or older (Table 1). 28% of staff had worked for less than 5 years and 33% had worked for 20 years or more in their current profession.

**Table 1:** Gender a﻿nd age group for 278 respondents split by main role

	Nursing/ Midwifery n = 86	Medical n = 34	AHPs n = 58	Other Therapeutic n = 39	Admin/ Support n = 61	Total n = 278
Gender:						
Female	72	21	46	26	49	214
Male	10	11	8	12	9	50
Missing	4	2	3	0	1	10
Age group:						
16–29	8	4	5	7	4	28
30–39	21	6	12	12	12	63
40–49	23	11	18	8	13	73
50–59	23	11	14	7	17	72
60+	4	2	4	4	13	27
Missing	7	0	4	0	0	11
Years in Profession:						
4	26	5	6	18	23	78
5–9	11	5	12	5	14	47
10–14	14	6	6	3	7	36
15–19	5	3	6	5	4	23
20+	30	15	27	7	11	90
Missing	0	0	1	1	2	4
Research as part of role ^a^	22 (26%)	12 (35%)	17 (29%)	18 (46%)	9 (15%)	78^c^ (100%)
Research active ^b^	19 (22%)	17 (50%)	16 (28%)	19 (49%)	7 (11%)	78^d^ (100%)

a: difference in proportions statistically significant P = 0.012

b: difference in proportions statistically significant P < 0.001

c: 9 cases missing staff group

d: 5 cases missing for staff group

Staff were categorised into 5 main roles: Nursing/Midwifery, AHPs, Medical/Dental, Other Therapeutic (comprising Healthcare Sciences, Medical Support, Other Therapeutic, Pharmacy and Psychology), Administration/Support Services (comprising Administration Services, Senior Managers and Support Services).

Overall, 87/315 staff (28%) stated that research related activities were part of their role description, though only 54 (62%) were currently research-active. Details of their staff grouping were available for 78 of these 87 respondents (Table 1). The proportion of staff with research as part of their role description differed significantly between the groups (Chi-square = 12.9, 4 degrees of freedom (df), P = 0.012). Respondents were asked what provisions were made to conduct research as part of their role. Of these 87 respondents the listed resources included library access (42%), time (39%), training (33%), software (29%), admin support (21%), research supervision (15%) and research funds (9%). The value of 42% for library access was at variance with the service which is freely available to all staff.

Furthermore, 83/305 staff (27%) were currently research-active, or had been research-active in the past 12 months though only 65% had research written into their role description. The proportion of research-active staff differed significantly between the staff groups (Chi-square = 26.2, 4 df, P < 0.001, Table 1). Ten staff (12%) had secured research funding, 15 (18%) had presented findings at a conference and 32 (39%) had co-authored a paper for publication in the previous 12 months.

Of the 503 respondents 124 (25%) had a postgraduate qualification with 26 (5%) having a PhD or MD. Twenty-four respondents were currently enrolled in a higher degree study or other professional development involving a research component (5 certificate, 7 undergraduate, 12 Postgraduate including 5 PhD/MD).

### The Research Capacity and Culture tool – Organisation level

The results for the 18 statements relating to aspects of research support by the organisation are listed in Table 2 (n = 501). The findings should be interpreted in relation to the percentage of ‘unsure’ responses which varied from 27.6 to 61.7% and was above 50% for 7 of the 18 items; the largest percentage was associated with the statement ‘The organisation has software programs for analysing research data’. Overall, median scores were greatest for the statements ‘The organisation promotes clinical practice based on evidence’, ‘supports the peer-reviewed publication of research’, ‘has identified experts accessible for research advice’, ‘ensures organisation planning is guided by evidence’, and ‘encourages research activities relevant to practice’. The lowest median score of 4 was for the statement ‘The organisation ensures staff career pathways are available in research’.

**Table 2:** Summary Statistics for Organisational Level Statements (n = 501) listed by Median, then by percentage ‘Unsure’

Qu	The Organisation:	Unsure %	n	Median	IQR
9	promotes clinical practice based on evidence	27.6	365	8	6–9
18	supports the peer-reviewed publication of research	53.4	235	7	3–9
13	has identified experts accessible for research advice	52.8	239	7	4–9
6	ensures organisation planning is guided by evidence	39.3	306	7	5–8
10	encourages research activities relevant to practice	35.3	326	7	4–8
12	has mechanisms to monitor research quality	57.3	216	6	3.25–8
7	has consumers involved in research	52.7	239	6	3–8
16	engages external partners (e.g. universities) in research	49.7	254	6	4–9
3	has a plan or policy for research development	48.1	262	6	4–8
14	supports a multi-disciplinary approach to research	44.9	278	6	4–8
15	has regular forums/bulletins to present research findings	43.1	287	6	3–8
1	has adequate resources to support staff research training	41.1	297	6	4–8
4	has senior managers that support research	38.5	310	6	4–8
11	has software programs for analysing research data	61.7	194	5	2–8
8	accesses external funding for research	55.3	226	5	3–8
17	supports applications for research scholarships/ degrees	55.0	229	5	3–8
2	has funds, equipment or admin to support research activities	44.1	282	5	3–7
5	ensures staff career pathways are available in research	48.3	261	4	2–6

These general findings were reflected in the 278 replies for which we had recordings of the staff main roles (Additional File 1). For example, all 5 staff categories recorded high median scores and generally low rates of ‘unsure’ responses associated with the statement concerning the organisation promoting clinical practice based on evidence. Again, low median scores were noted for the statement ‘The organisation ensures staff career pathways are available in research’.

A Kruskal-Wallis test was undertaken to compare the distribution of scores for each statement across the different main roles. The only statistically significant difference between main roles was for the statement ‘The organisation ensures planning is guided by evidence’ (P = 0.002). There were significant differences after a Bonferroni correction for multiple comparisons between the medians recorded for the ‘Other therapeutic group’ (median 8) and the Admin/Support Services (median 5, P = 0.009), Medical/Dental (median 6, P = 0.028) and AHPs (median 5, P = 0.012).

### The Research Capacity and Culture tool – Team level

The results for the 19 statements relating to aspects of research support by the respondent’s team are listed in Table 3 (n = 151). The percentage of unsure responses varied from 14.6 to 45.7% and was least for the statement ‘The team has team leaders that support research’, for which the median score was 8, and was greatest for the statement ‘The team has software available to support research activities’, for which the median score was 4. High median scores were associated with statements that reflected support for research within the team and research activities related to practice. Low median scores were associated with practical aspects such as provision of incentives and software for research.

**Table 3:** Summary Statistics for Team Level Statements (n = 151) listed by Median, then by percentage ‘Unsure’

Qu	The Team:	Unsure %	n	Median	IQR
18	supports peer-reviewed publication of research	35.8	97	8	3–9
7	does planning that is guided by evidence	20.5	120	8	5–10
10	conducts research activities relevant to practice	19.9	121	8	5–10
5	has team leaders that support research	14.6	129	8	5–9
15	supports a multi-disciplinary approach to research	24.5	114	7.5	4.75–9
12	has mechanisms to monitor research quality	38.4	93	7	3–9
8	has consumer involvement in research activities/planning	33.1	101	7	3.5–9
17	has external partners (e.g. universities) engaged in research	31.1	104	7	3–9
13	has identified experts accessible for research advice	26.4	111	7	4–9
14	disseminates research results at research forums/seminars	30.4	105	6	4–9
1	has adequate resources to support staff research training	19.9	121	6	3–8
4	ensures staff involvement in team level planning for research development	19.9	121	6	3–8
6	provides opportunities to get involved in research	15.2	128	6	4–9
9	has applied for external funding for research	43.0	86	5	1.75–8.25
11	supports applications for research scholarships/ degrees	38.4	93	5	3–9
2	has funds, equipment or admin to support research activities	23.8	115	5	2–8
3	does team level planning for research development	19.2	122	5	2–8
19	has software available to support research activities	45.7	82	4	1–8
16	has incentives & support for mentoring activities	38.4	93	4	2–8

When the data were subdivided by main role there were some particularly marked differences between staff categories (Additional File 2). On average, the nursing/midwifery group median scores were higher than those for the other groups, with the AHPs scoring particularly low for most statements (e.g. lowest median for 9 of the 19 statements). However, the number of individual responses per main role was relatively small for those questions with a relatively large percentage of ‘unsure’ responses.

A Kruskal-Wallis test was undertaken to compare the distribution of scores for each statement across the different main roles. The only statistically significant difference between main roles was for the statements ‘The team has consumer involvement in research activities/planning’ (P = 0.010) and ‘The team has software available to support research activities’ (P = 0.007). The median score recorded for the statement ‘The team has consumer involvement in research activities / planning’ was 8 for the Nursing/Midwifery group and this was significantly greater than that for the Medical/Dental group (median 5, P = 0.039) and the AHPs (median 5, P = 0.021), after Bonferroni correction. The median score for the statement ‘The team has software available to support research activities’ was 8 for the Nursing/Midwifery group and this was significantly greater than that for the AHPs (median 1.5, P = 0.008) after Bonferroni correction.

### Barriers and motivators to research in the team

Free-text responses were provided by 102 staff for which the greatest barrier was a lack of time, mentioned by 72 (70%). This was the principal barrier mentioned by 38 of the 54 respondents (70%) who had research as part of their role description. Other issues raised were clinical workload, pressure to reduce patient waiting times, and lack of funds, backfill and capacity. Several responses referred to a lack of encouragement and support by managers and the impact of SARS-CoV-2 where staff had been redeployed and clinical priorities changed in response to the pandemic.

Principal motivators cited in 103 responses referred to improving practice and patients’ experience / outcomes. Other motivators were finding new treatments, career development, retaining staff and self-esteem.

### The Research Capacity and Culture tool – Individual success or skill level

The results for the 14 statements relating to personal aspects of research are listed in Table 4 (n = 315). The percentage of unsure responses was, in general, less than those noted in the organisation and team questions and varied from 11.7 to 24.4%. Overall, high skill levels were cited for finding and reviewing the literature. Lower skill levels were associated with preparing research grants, ethics applications, protocols, journal articles and providing advice for less experienced researchers. Nurses, midwives, AHPs and Admin/Support services staff reported low levels of skill for preparing reports, analysing data, both qualitative and quantitative, and providing advice to less experienced researchers (Additional File 3). Medical/Dental and Other therapeutic staff reported moderate skill levels in writing for publication and in providing advice to less experienced researchers. All groups reported low levels of skills in securing research funding, moderate levels of skill in designing questionnaires and high skill levels in collecting data, finding and reviewing the literature.

**Table 4:** Summary Statistics for Individual Level Statements (n = 315) listed by Median, then by percentage ‘Unsure’

Qu	Own Success or Skill Level:	Unsure %	n	Median	IQR
1	Finding relevant literature	12	278	8	5–8
2	Critically reviewing the literature	14	270	7	4–8
8	Collecting data e.g. surveys, interviews	16	265	6	3–8
11	Analysing quantitative research data	21	249	5	2–8
7	Designing questionnaires	17	260	5	2–8
10	Analysing qualitative research data	21	250	4	2–7
12	Writing a research report	21	249	4	2–8
3	Using a computer referencing system (e.g. Endnote)	20	251	4	1–7
9	Using computer data management systems	18	257	4	2–7.5
4	Writing a research protocol	20	252	3	1–6
6	Submitting an ethics application	24	240	2	1–6
13	Writing for publication in peer-reviewed journals	24	238	2	1–6
14	Providing advice to less experienced researchers	23	243	2	1–5
5	Securing research funding	24	240	1	1–3.75

### Personal barriers and motivators to research

Responses were received from 282 respondents. The top 5 barriers were other work roles taking priority, lack of time, the desire for work / life balance, lack of research skills and lack of suitable backfill (Table 5). Additional, relevant comments received from 12 respondents mentioned, for example, lack of nursing staff, research nurse team capacity, the research approval process, the problem of being on a rolling contract and the need (for one respondent) to take a pay cut to work in a full-time research post. Overall, 11% were not interested in research.

**Table 5:** Personal Barriers to do research

Barrier	n	Percent of 282
Other work roles take priority	183	64.9
Lack of time for research	171	60.6
Desire for work / life balance	108	38.3
Lack of skills for research	97	34.4
Lack of suitable backfill	79	28.0
Intimidated by fear of getting it wrong	76	27.0
Other personal commitments	71	25.2
Lack of funds for research	70	24.8
Lack of admin support	63	22.3
Intimidated by research language	56	19.9
Lack of a co-ordinated approach to research	51	18.1
Lack of support from management	48	17.0
Lack of software for research	44	15.6
Lack access to equipment for research	39	13.8
Isolation	32	11.3
Not interested in research	31	11.0
Lack of library/internet access	14	5.0

The top 5 motivators were to develop skills, increase job satisfaction, keep the brain stimulated, a problem identified that needs changing and career advancement (Table 6). Additional, relevant motivators were received from 19 respondents, 13 of whom mentioned “to improve patient care”. Overall, 47/282 (16%) were not motivated to do research; the proportion for each staff group was Admin/support services 25%, Nursing/midwifery 23%, Medical/Dental 9%, AHPs 7% and Other therapeutic 5%.

**Table 6:** Personal Motivators to do research

Motivator	n	Percent of 282
To develop skills	144	51.1
Increased job satisfaction	126	44.7
To keep the brain stimulated	111	39.4
Problem identified that needs changing	110	39.0
Career advancement	94	33.3
Opportunities to participate at own level	81	28.7
Desire to prove a theory / hunch	79	28.0
Dedicated time for research	65	23.0
Increased credibility	61	21.6
Mentors available to supervise	54	19.1
Research encouraged by managers	53	18.8
Links to Universities	53	18.8
Not interested to do research	47	16.7
Research written into role description	40	14.2
Colleagues doing research	37	13.1
Forms part of post-graduate study	35	12.4
Grant funds	29	10.3
Study or research scholarships available	24	8.5

Respondents were asked ‘As a result of the pandemic has your attitude to research in the NHS changed?’ to which 171/503 (34%) had changed their attitude. An analysis confined to the 278 respondents who indicated their main role revealed the largest proportion was for the Admin and Support Services (44%) though, overall, the proportions did not differ significantly between the different staff groups (Chi-square = 4.9, df = 4, P = 0.30).

Free-text comments on how their attitude had changed identified the following 6 themes: (1) development of vaccines and new treatments, (2) engagement in Covid studies and increased willingness to volunteer as a participant in research studies, (3) the importance of Research and Development in the NHS and the need to expand it, (4) the impact of the pandemic on services, (5) the experience of working in the NHS throughout the pandemic and (6) taking pride in the service.

Respondents were asked “as a result of the pandemic has your attitude to volunteering for a research study changed?” Almost 60% of 502 respondents had not changed their attitude to volunteering for a research study as a result of the pandemic, with 53% stating they would always consider volunteering. Of the 205 respondents who had changed their attitude to volunteering 92% agreed that they were now more likely to volunteer for a research study.

Respondents were asked for suggestions to increase research activity in the NHS. Six themes emerged: (1) Increase the profile of research within the Health Board by engaging with staff and the public, advertising for participants, offering incentives and distributing regular updates on research activity, (2) Increase the organisation’s engagement with departments to promote research and to encourage research as part of a career pathway, (3) Increase resources such as funding, training in research skills, (4) Improve the research experience, reduce bureaucracy through practical support with preparing grants, protocols, reports etc., (5) Change work patterns to allow protected time and backfill for research, and (6) Foster links with academia.

Respondents were asked to complete the statement: “I would be more likely to get involved with undertaking or supporting research, if…” Responses suggested that staff were more likely to get involved in research if they had the motivation (interest), the time to do it, the training needed, support from the organisation and awareness of what studies were available. Some staff already research-active were struggling to cope with their competing workload.

## Discussion

The UK’s NHS seeks to improve the health of the population by providing clinical services relevant to its need. The recent pandemic has stretched the service considerably leaving staff under pressure to provide an appropriate service to meet the population’s health needs resulting in an inevitable extension of waiting lists. Despite these challenges the NHS remains committed to its culture of research and is seeking to expand its research activities through an increase in capacity [1, 2, 14]. The development of a research culture with provision of appropriate resources is fundamental to achieve this goal and requires investment at the level of organisation, team and individual [15]. Our study has revealed a commitment for promoting evidence-based practice and supporting research at both organisational and team levels though access to resources such as equipment, admin, software and funding was seen as a barrier amongst teams. We noted some marked differences in team responses with nurses/midwives returning higher median scores than AHPs in particular. A low score was reported for the organisation ensuring that staff career pathways are available in research. Respondents rated personal skill levels as high for finding and critically reviewing literature but low for preparing research grants, ethics applications, protocols and journal articles. However, in general, medical and other therapeutic staff reported higher levels of skills at these practical aspects of research compared with nurses, midwives, AHPs and Admin staff.

Barriers to research at both team and personal levels related mainly to lack of time, lack of backfill and pressure of clinical work to reduce patient waiting times. Principal motivators were to improve personal skills, increase job satisfaction, improve practice and patient experience and to promote career development.

About a third of respondents had changed their attitude to research in the NHS as a result of the pandemic. This was most marked in the admin and support services where 44% reported a positive change in attitude. About 40% of 502 respondents had changed their attitude to volunteering for a research study with 92% of them stating they were now more likely to participate. Respondents were now more aware of the importance of research in the rapid development of vaccines and new treatments. Also, they recognised the importance of participating in research both as a volunteer and active researcher. Some respondents expressed pride in working for the NHS and the contribution made by it in the global effort to combat SARS-CoV-2.

Eighty-three staff had been research-active in the previous 12 months. The proportions differed significantly between staff groups with relatively more medical and therapeutic staff research-active compared with nurses, midwives, AHPs and Admin staff (Table 1). Many live research studies in the Health Board were suspended during the pandemic as research activity switched to new Covid-related studies. However, research-active staff maintained their activity during this time in reporting findings in journal articles and conference presentations. In addition, 10 staff had secured research funding.

Suggestions to increase research activity included the adoption of policies by the organisation to increase the profile of current research opportunities (for both staff and patient volunteers), to provide protected time, to increase resources (including funding and training) and to reduce bureaucracy. The latter was particularly mentioned by some research-active staff who were already struggling with their competing workload.

Our findings for research capacity at organisation, team and individual levels are roughly in accordance with previous studies using the RCCT from the health sector in Australia [4, 5, 16] and for AHPs from the UK NHS [17]. Cordrey and colleagues [17] reported results from 93 AHPs working in a hospital in Oxford. They also noted strengths in organisation and team levels in promoting clinical practice based on evidence and weaknesses in providing resources to support research activity such as training, software, admin, funds and equipment, similar to our findings (see Additional Files 1 and 2).

Common barriers to research cited in the published literature include lack of time and funds [4, 5, 12, 16, 18–20], lack of backfill and pressure of clinical work [4, 8, 19, 21], all of which were also cited as principal barriers in our study. Finding time to devote to research is a challenge for busy clinicians under pressure to manage their clinical loads. In our study 87 respondents had research as part of their role description but only 39% had time allocated for the activity. Wenke et al. [7] evaluated a short-term funding initiative to provide 16 AHPs with back fill to allow them to undertake research activities, including preparing ethics applications, collecting data, writing up and submitting journal articles for peer-review. Each AHP had support from a research fellow and was able to generate research outputs over the 4-week, full-time equivalent funded period. The AHPs reported improvements in scores for self-efficacy and the development of skills many of which were statistically increased.

A regular concern reported by others is the lack of career pathways for research-interested health professionals [18, 22] and this was scored lowest in the organisation domain in our study. However, not all NHS staff are interested in, or suited to, research; in our study 16.7% of respondents were unmotivated to engage in research though we would argue that research-awareness is important for all grades of staff engaged in clinical duties. To this end it was encouraging that respondents reported good skill levels in finding and interpreting the literature.

Published studies on ways to increase research capacity in the health sector have reported the need to adopt a ‘whole systems’ approach [9, 18, 21, 23]. This includes valuing research as core business [1, 8, 14, 21] and encouraging senior management to incorporate it into strategic plans [9, 13, 18]. Strong leadership and motivation at team level is also considered critical [20, 24] as are personal skills with practical administrative and academic support through the employment of, or access to research facilitators [9, 11–13], librarians [9] and the provision of protected time and backfill [11]. Strong links sharing resources with external partners, including academia, is also considered favourable in developing a research culture [9, 10, 13, 18, 19].

The personal research capacity differs between professional groups [4, 5, 19] depending on their exposure to research in their professional training. Psychologists, for example, compared with other health professionals, have research firmly embedded in their doctoral-level professional development [19]. Accordingly, different approaches, or the same approach with differing degrees of engagement, may be required to increase interest in research within disparate health professions [5].

### Strengths and limitations

The low, overall response rate of 5.5% is consistent with response rates for similar surveys in different heath settings [4, 5] and is a conservative estimate given the denominator was the total number of staff (9,145). It was not possible to send individual invitations to each staff member so the denominator is based on the assumption that all staff saw the invitation on central platforms and could have responded. In reality, this is unlikely. We acknowledge that our findings, based on a 5.5% response rate, may not be considered representative of the situation in other Scottish Health Boards, though our survey was intended mainly as a prerequisite to the development of local strategies to increase research activity in our Health Board. Our sample may be biased as the invitation to take the survey would more likely be accepted by staff members already interested in or active in research. For example, the proportion of staff unmotivated to engage in research varied between staff groups with about a quarter of nurses/midwives and Admin/support staff being uninterested. Furthermore, about half of the medical/dental and other therapeutic groups were research-active; the equivalent proportions were 28% for AHPs, 22% for nurses/midwives and 11% for Admin/Support services.

We used a validated questionnaire that has been used in other studies exploring the research culture and capacity in health care organisations from Australia [4, 5, 7, 16, 20, 23, 25] and in the UK NHS [17, 22]. The tool seeks to measure research capacity using a holistic approach including the roles of the organisation, team and individual, with additional data collected on the barriers and motivators at team and personal levels. Hence our findings may be compared directly with other studies using the same approach. However, the proportion of responses that were scored ‘unsure’ was relatively high for some questions, challenging the validity of our interpretation of the summary scores and their comparison with other studies using the same questionnaire.

To our knowledge this is the first survey of the research capacity and culture of a Scottish Health Board, using the RCCT validated instrument, and the first enquiry into changes in attitude to research amongst NHS staff arising from the pandemic. The latter findings are encouraging in light of the UK and Scottish Government’s visions on increasing research awareness and activity in the NHS [1, 14].

### Recommendations

Our Health Board’s Research, Innovation and Knowledge (RIK) Department has a director of research providing strategic leadership and liaison with senior management, an assistant director, a senior research adviser providing academic and statistical support, librarians, 21 research nurses and an admin team providing research governance support with management approvals, ethics applications, quality assurance and finance matters. Resources include a clinical research facility and an education programme covering research skills. Staff taking post-graduate courses that entail a research project are encouraged to seek advice early on in their course to take advantage of the resources available.

Amongst Scotland’s Health Boards four are large, Nodal Boards (Teaching Hospital status) with strong links with academia and, accordingly, large research outputs. By comparison, NHS Fife is one of Scotland’s smaller, non-Nodal Health Boards but, in terms of research activity (for example, study recruitment), it lies second amongst the remaining eight research-active Health Boards. The number of research projects registered across our Health Board was 237 in 2018-19 and 259 in 2019-20. This dropped to 103 in 2020-21 as studies were suspended due to the pandemic and activity switched to Covid-related studies. Currently, for 2021-22 there are 98 studies registered so activity is still below pre-pandemic levels.

Our findings suggest that a significant intervention in promoting further research will require the provision of protected time, backfill and funding, particularly for pilot studies. Ideally, research should be core business for the organisation with further initiatives directed at middle and team management to promote research as a team activity. However, clinical workload may impinge on uptake in the short-term as the health service recovers from the impact of the pandemic on patient services.

The Scottish Government provides research training fellowships that are open to all clinical staff grades in the NHS in an open-bidding process. However, it is recognised that the number of successful applications from non-medical staff are very low leading to disenchantment with the scheme by some staff grades, though efforts are under way to address these concerns [14]. One challenge is to make research part of the career development of all interested clinical staff, requiring commitment by senior and middle management to include it in personal development plans. Another approach is to encourage any healthcare staff who have a role in a research team, but who are not included on a professional register, to join the Directory of Clinical Research Practitioners (CRP), established in September 2018, and, subsequently, the Accredited Register of CRPs, established in March 2021 [2, 26]. Advantages include career development and increased professional credibility through inclusion on an accredited professional register.

The findings from the present study also suggest there is need to make it easier, at organisational, team and individual levels, to engage in research by increasing resources to provide funds and practical support to reduce bureaucracy and assist in the preparation of protocols and ethics applications etc. This latter requirement is in line with one of the seven action points featured in the Government’s strategy which seeks to streamline the process of setting up a study [1, 2]. These approaches may then enable those staff who express an interest in research, but who lack the necessary skills, resources and time, to become research-active, thereby increasing the Board’s overall research capacity.

Finally, our findings will be shared with other Health Boards in Scotland to allow them to develop local initiatives to expand their own research capacity and culture.

## Conclusion

Our survey has revealed a positive change in attitude to research amongst NHS staff arising from the SARS-CoV-2 pandemic and quantified the level of interest in and activity in research that will provide a baseline against which any initiatives introduced to increase research capacity and capability can be compared. We identified issues over the need for protected time, funding, training in all aspects of the research process, support to complete documents (protocols, ethics forms, grant applications), reduced bureaucracy, support and encouragement by managers (senior, middle and team), research embedded for career advancement, research made part of everyday practice, increased awareness raising of research activity and opportunities for patients and staff. The challenges are considerable particularly as the NHS recovers from the hiatus in its usual service delivery that resulted from the pandemic. To increase capacity will require top-down as well as bottom-up initiatives to create a supportive environment where research can flourish and novice researchers can develop the skills needed to initiate and sustain their own research career.

## Electronic supplementary material

Below is the link to the electronic supplementary material.

Supplementary Material 1

Supplementary Material 2

Supplementary Material 3

## Data Availability

All data generated or analysed during this study are included in this published article and supplementary information files.

## Declarations

### Approvals

Management approval was obtained from NHS Fife. The study protocol was reviewed and approved by internal staff in NHS Fife and by one external reviewer from NHS Forth Valley. A formal review by an NHS ethics committee was not required following advice obtained using the Health Research Authority decision tools (www.hra-decisiontools.org.uk) and directly from the East of Scotland Research Ethics Service (NHS Tayside).
