# Supplementary material for: The research interest, capacity and culture of NHS staff in South East Scotland and changes in attitude to research following the pandemic: a cross-sectional survey
Source: BMC Health Serv Res. 2023 Mar 7;23:220. doi: 10.1186/s12913-023-09196-y (PMC9990035; doi:10.1186/s12913-023-09196-y)
Supplement: Supplementary file 1 — Supplementary Material 1 [file 12913_2023_9196_MOESM1_ESM.docx]

**Additional File 1. RCCT responses by Main Role, Organisation Level**

| Qu | The Organisation: | Nursing/Midwifery  n=86 | | AHP  n=58 | | Medical/Dental  n=34 | | Other Therapeutic  n=39 | | Admin/Support Services n=61 | |
| --- | --- | --- | --- | --- | --- | --- | --- | --- | --- | --- | --- |
|  |  | Unsure (%) | Median | Unsure (%) | Median | Unsure (%) | Median | Unsure (%) | Median | Unsure (%) | Median |
| 1 | has adequate resources to support staff research training | *43* | 6 | *38* | 5 | *35* | 5.5 | *33* | 6 | *54* | 6.5 |
| 2 | has funds, equipment or admin to support research activities | *50* | 5 | *38* | 4 | *44* | 5 | *38* | 5 | *52* | 4 |
| 3 | has a plan or policy for research development | *50* | 6 | *43* | 7 | *56* | 7 | *46* | 6 | *48* | 6 |
| 4 | has senior managers that support research | *38* | 7 | *26* | 6 | *47* | 5.5 | *33* | 6.5 | *44* | 7 |
| 5 | ensures staff career pathways are available in research | *42* | 5 | *36* | 3 | *56* | 4 | *54* | 4.5 | *57* | 4 |
| 6 | ensures organisation planning is guided by evidence | *34* | 8 | *28* | 5 | *41* | 6 | *36* | 8 | *57* | 5 |
| 7 | has consumers involved in research | *57* | 6 | *36* | 7 | *74* | 6 | *54* | 5 | *56* | 6 |
| 8 | accesses external funding for research | *59* | 7 | *53* | 5 | *59* | 7.5 | *51* | 6 | *57* | 6 |
| 9 | promotes clinical practice based on evidence | *26* | 8 | *12* | 8 | *29* | 8 | *23* | 9 | *44* | 8 |
| 10 | encourages research activities relevant to practice | *35* | 7 | *22* | 5 | *41* | 7 | *28* | 6.5 | *51* | 7.5 |
| 11 | has software programs for analysing research data | *66* | 7 | *48* | 5 | *79* | 5 | *56* | 7 | *59* | 7 |
| 12 | has mechanisms to monitor research quality | *59* | 7 | *53* | 6 | *59* | 7.5 | *59* | 7 | *62* | 8 |
| 13 | has identified experts accessible for research advice | *56* | 8 | *41* | 7 | *59* | 6 | *46* | 7 | *54* | 6 |
| 14 | supports a multi-disciplinary approach to research | *52* | 8 | *34* | 6 | *50* | 7 | *36* | 6 | *52* | 7 |
| 15 | has regular forums/bulletins to present research findings | *43* | 7 | *36* | 7 | *38* | 8 | *46* | 5 | *46* | 6 |
| 16 | engages external partners (eg universities) in research | *57* | 7 | *43* | 7 | *59* | 8 | *38* | 6 | *44* | 7 |
| 17 | supports applications for research scholarships/ degrees | *57* | 6 | *41* | 5 | *62* | 8 | *54* | 5 | *59* | 5 |
| 18 | supports the peer-reviewed publication of research | *57* | 7 | *43* | 5 | *59* | 8 | *41* | 6 | *59* | 8 |
