# Supplementary material for: The research interest, capacity and culture of NHS staff in South East Scotland and changes in attitude to research following the pandemic: a cross-sectional survey
Source: BMC Health Serv Res. 2023 Mar 7;23:220. doi: 10.1186/s12913-023-09196-y (PMC9990035; doi:10.1186/s12913-023-09196-y)
Supplement: Supplementary file 2 — Supplementary Material 2 [file 12913_2023_9196_MOESM2_ESM.docx]

**Additional File 2. RCCT responses by Main Role, Team Level**

| Qu | The Team: | Nursing/Midwifery  n=35 | | AHP  n=28 | | Medical/Dental  n=21 | | Other Therapeutic  n=24 | | Admin/Support Services n=18 | |
| --- | --- | --- | --- | --- | --- | --- | --- | --- | --- | --- | --- |
|  |  | Unsure (%) | Median | Unsure (%) | Median | Unsure (%) | Median | Unsure (%) | Median | Unsure (%) | Median |
| 1 | has adequate resources to support staff research training | *20* | 7.5 | *11* | 3 | *10* | 5 | *21* | 5 | *39* | 5 |
| 2 | has funds, equipment or admin to support research activities | *31* | 8 | *14* | 2 | *14* | 5.5 | *25* | 5 | *33* | 4 |
| 3 | does team level planning for research development | *23* | 7 | *7* | 3.5 | *14* | 6 | *21* | 5 | *33* | 7 |
| 4 | ensures staff involvement in developing that plan | *17* | 8 | *14* | 4.5 | *19* | 6 | *25* | 5 | *33* | 6 |
| 5 | has team leaders that support research | *17* | 8 | *4* | 7 | *0* | 7 | *17* | 8 | *33* | 6.5 |
| 6 | provides opportunities to get involved in research | *17* | 7 | *4* | 6 | *0* | 7 | *21* | 6 | *33* | 5 |
| 7 | does planning that is guided by evidence | *17* | 8 | *14* | 8 | *24* | 7.5 | *17* | 8.5 | *33* | 5.5 |
| 8 | has consumer involvement in research activities/planning | *34* | 8 | *21* | 5 | *38* | 5 | *29* | 5 | *56* | 5.5 |
| 9 | has applied for external funding for research | *43* | 8 | *32* | 3 | *33* | 2.5 | *42* | 5 | *44* | 6 |
| 10 | conducts research activities relevant to practice | *26* | 8.5 | *7* | 7 | *10* | 9 | *25* | 8.5 | *39* | 5 |
| 11 | supports applications for research scholarships/ degrees | *40* | 7 | *25* | 5 | *33* | 4.5 | *46* | 5 | *61* | 7 |
| 12 | has mechanisms to monitor research quality | *34* | 8 | *39* | 3 | *24* | 7 | *38* | 5 | *50* | 7 |
| 13 | has identified experts accessible for research advice | *29* | 8 | *18* | 6 | *5* | 7.5 | *29* | 8 | *44* | 7 |
| 14 | disseminates research results at research forums/seminars | *31* | 7 | *21* | 5 | *10* | 8 | *25* | 6.5 | *50* | 7 |
| 15 | supports a multi-disciplinary approach to research | *29* | 9 | *21* | 7.5 | *0* | 7 | *21* | 7 | *44* | 6 |
| 16 | has incentives & support for mentoring activities | *37* | 6.5 | *25* | 3 | *33* | 2 | *33* | 5 | *61* | 5 |
| 17 | has external partners (eg universities) engaged in research | *34* | 9 | *21* | 3 | *19* | 8 | *25* | 6.5 | *50* | 7 |
| 18 | supports peer-reviewed publication of research | *37* | 8 | *21* | 6.5 | *29* | 8 | *38* | 8 | *56* | 5.5 |
| 19 | has software available to support research activities | *43* | 8 | *43* | 1.5 | *48* | 2 | *42* | 5 | *50* | 3 |
