# Supplementary material for: The research interest, capacity and culture of NHS staff in South East Scotland and changes in attitude to research following the pandemic: a cross-sectional survey
Source: BMC Health Serv Res. 2023 Mar 7;23:220. doi: 10.1186/s12913-023-09196-y (PMC9990035; doi:10.1186/s12913-023-09196-y)
Supplement: Supplementary file 3 — Supplementary Material 3 [file 12913_2023_9196_MOESM3_ESM.docx]

**Additional File 3. RCCT responses by Main Role, Individual success or skill level**

| Qu | Own Success or Skill Level: | Nursing/Midwifery  n=86 | | AHP  n=58 | | Medical/Dental  n=34 | | Other Therapeutic  n=39 | | Admin & Support Services n=61 | |
| --- | --- | --- | --- | --- | --- | --- | --- | --- | --- | --- | --- |
|  |  | Unsure % | Median | Unsure % | Median | Unsure % | Median | Unsure % | Median | Unsure % | Median |
| 1 | Finding relevant literature | *12* | 8 | *3* | 7 | *0* | 8 | *8* | 8 | *26* | 6 |
| 2 | Critically reviewing the literature | *15* | 7 | *5* | 6 | *0* | 8 | *8* | 8 | *28* | 5 |
| 3 | Using a computer referencing system (eg Endnote) | *22* | 5 | *17* | 2 | *9* | 5 | *13* | 5 | *31* | 3.5 |
| 4 | Writing a research protocol | *23* | 4 | *14* | 2 | *3* | 4 | *10* | 6 | *34* | 2 |
| 5 | Securing research funding | *30* | 2 | *14* | 1 | *3* | 2 | *15* | 2 | *36* | 1 |
| 6 | Submitting an ethics application | *31* | 2 | *14* | 1 | *12* | 3 | *18* | 5 | *33* | 2 |
| 7 | Designing questionnaires | *21* | 5 | *9* | 5 | *6* | 5 | *15* | 7 | *26* | 7 |
| 8 | Collecting data e.g. surveys, interviews | *21* | 6 | *7* | 5.5 | *3* | 7 | *10* | 8 | *28* | 8 |
| 9 | Using computer data management systems | *26* | 5 | *10* | 3 | *6* | 3 | *15* | 5 | *26* | 7 |
| 10 | Analysing qualitative research data | *27* | 3 | *5* | 4 | *6* | 4.5 | *15* | 6 | *31* | 5 |
| 11 | Analysing quantitative research data | *28* | 3 | *5* | 4 | *6* | 5.5 | *15* | 7 | *31* | 5 |
| 12 | Writing a research report | *30* | 3 | *7* | 3.5 | *6* | 6.5 | *13* | 8 | *31* | 4 |
| 13 | Writing for publication in peer-reviewed journals | *34* | 2 | *14* | 2 | *6* | 6 | *21* | 5 | *33* | 1 |
| 14 | Providing advice to less experienced researchers | *33* | 2 | *7* | 2 | *6* | 5 | *23* | 4.5 | *33* | 2 |
